# Multiplex PCR for identification of two butterfly sister species: *Eurema mandarina* and *Eurema hecabe*

**DOI:** 10.1186/s13104-020-05093-3

**Published:** 2020-05-27

**Authors:** Mai N. Miyata, Daisuke Kageyama, Masashi Nomura

**Affiliations:** 1 Graduate School of Horticulture, Chiba University, Matsudo, Chiba 271-8510 Japan; 2 Institute of Agrobiological Sciences, National Agriculture and Food Research Organization, 1-2 Owashi, Tsukuba, Ibaraki 305-0851 Japan

**Keywords:** *Eurema mandarina*, *Eurema hecabe*, *Eurema blanda*, Multiplex PCR

## Abstract

**Objective:**

In insects, closely related species are often difficult or impossible to distinguish solely by morphological traits. Mitochondrial DNA (mtDNA) markers are often useful and reliable for distinguishing closely related species. However, useful mtDNA markers can be unavailable, particularly when such species pairs experienced hybrid introgression in the past. Although polymorphic nuclear DNA markers would be necessary to distinguish such species pairs, recombination, multiple copies, and slower mutation rates of the nuclear DNA compared with those of mtDNA often make it challenging. The objective of this study was to develop a multiplex polymerase chain reaction that can reliably amplify and distinguish the *Tpi* sequences of *Eurema mandarina* and *Eurema hecabe*.

**Results:**

We successfully analyzed the nucleotide sequences of the Z chromosome-linked triose phosphate isomerase (*Tpi*) gene to develop a multiplex polymerase chain reaction (PCR) that amplified ca. 120-bp products for *E. mandarina* and ca. 375-bp products for *E. hecabe*. We suggest that multiplex PCR using *Tpi* with appropriately designed primers can be used to accurately and reliably distinguish between other closely related Lepidoptera species.

## Introduction

Insects are the most abundant and diverse group of living organisms on this planet [1]. Some congeneric insect species, which were once considered to be the same species, have later be divided into distinct species based on detailed morphological characters or nucleotide sequences [2]. Mitochondrial DNA (mtDNA), such as cytochrome c oxidase subunit I (COI), is often used as a molecular marker to distinguish closely related species [3–5]. However, mtDNA markers cannot be used for species identification when closely related species have experienced hybrid introgression with each other in the past [6–10]. Although polymorphic nuclear DNA markers are necessary to distinguish such species [11], it is sometimes challenging to design appropriate primers for nuclear DNA because of the possibility of recombination and multiple copies.

Here, we focused on two sister species of butterfly: *Eurema mandarina* and *Eurema hecabe* (Lepidoptera; Pieridae). These species are very difficult to distinguish morphologically, and they were considered as a single species, *E. hecabe*, for a long time [12]. However, Kato and Handa (1992) found that temperate populations and subtropical populations of *E. hecabe* differed in their expression of polyphenism in response to photoperiod and temperature [13]. Following this discovery, it was found that the two types of *E. hecabe* were distinct in a number of traits, such as their host plants [14], wing fringe color [15], reflection pattern against ultra-violet rays [16], allelic frequencies of allozymes [17], and nuclear DNA sequences [18]. These data consistently and strongly suggest that the temperate populations with a yellowish wing fringe (Y type) and the subtropical populations with a brownish wing fringe (B type) constitute closely related but distinct biological species, *E. mandarina* and *E. hecabe*, respectively [19, 20]. Therefore, *E. mandarina* and *E. hecabe* are a good model system to investigate the evolutionary aspects of closely related species, such as their speciation process, adaptation to local environments, and biogeographical history [18, 21].

In both *E. mandarina* and *E. hecabe*, a single strain of *Wolbachia* endosymbiont, which causes cytoplasmic incompatibility and is referred to as *w*CI, is fixed in most of the populations of these two congeneric species. Based on molecular phylogenetic analyses, it has been suggested that the two species experienced hybrid introgression quite recently in the evolutionary timeframe: cytoplasm of *E. hecabe* was considered to have moved to *E. mandarina* together with *w*CI, and then *w*CI-occurring cytoplasm experienced a selective sweep within and across populations through the effect of cytoplasmic incompatibility [18]. Similar events of hybrid introgression have also been reported for other species [22, 23]. Therefore, *E. mandarin*a and *E. hecabe* cannot be distinguished using mtDNA [24].

According to Narita et al. (2006), nucleotide sequences of the Z chromosome-linked triose phosphate isomerase (*Tpi*) gene were distinct between *E. mandarina* and *E. hecabe* [18]. To avoid the complications of cloning and sequencing of the *Tpi* sequences, we developed a multiplex polymerase chain reaction (PCR) that reliably amplifies species-specific sequences of *Tpi* from *E. mandarina* and *E. hecabe*. This method allows easy and unambiguous identification of the two butterflies.

## Main text

### Materials and methods

#### Sample collection and morphological identification

The collection sites and number of *Eurema* individuals used in this study are listed in Table 1 (see Additional file 1 for details). We sampled 29 female and 38 male *E. hecabe* from 4 populations and 22 female and 16 male *E. mandarina* from 14 populations which were difficult to distinguish by morphological observation. Additionally, we sampled 6 females from 1 population of *E. blanda*, which is a species that is diverged from *E. hecabe* and *E. mandarina*. Wild-caught *E. hecabe* and *E. mandarina* were brought into the laboratory and carefully inspected under a dissecting microscope for morphological species identification using wing fringe color and cell spots on the underside of the forewing [25]. *E. blanda* specimens were easily identified by the black shape on a section of their forewings, shape of their hindwing, and three cell spots on the underside of their forewing [12], and they are morphologically distinct from *E. mandarina* and *E. hecabe*. After morphological species identification, all the samples were stored at − 30 °C until DNA extraction.

**Table 1:** Collection sites of the butterflies used in this study

Species	Location	No. of examined individuals
*Eurema hecabe*	Okinawa Is., Okinawa	8 (8f)
	Ishigaki Is., Okinawa	8 (8f)
	Yonaguni Is., Okinawa	47 (9f, 38m)
	Taiwan	4 (4f)
*Eurema mandarina*	Morioka, Iwate	3 (3f)
	Minamiuonuma, Nigata	2 (1f, 1m)
	Mashiko, Tochigi	1 (1f)
	Karuizawa, Nagano	3 (1f, 2m)
	Tsukuba, Ibaraki	3 (2f, 1m)
	Chichibu, Saitama	3 (3m)
	Matsudo, Chiba	3 (3f)
	Kimitsu, Chiba	1 (1f)
	Fujiyoshida, Yamanashi	3 (1f, 2m)
	Hokuto, Yamanashi	3 (1f, 2m)
	Shimanto, Kochi	3 (1f, 2m)
	Himeshima Is., Oita	3 (3m)
	Tanegashima Is., Kagoshima	5 (5f)
	Okinawa Is., Okinawa	2 (2f)
*Eurema blanda*	Ishigaki Is., Okinawa	6 (6f)

Numbers of females and males are in parentheses (f: females, m: males)

#### DNA extraction

A DNeasy Blood & Tissue Kit (QIAGEN, Tokyo, Japan) was used to extract DNA from all samples. From each individual butterfly, ca. 50 mg thoracic muscles were squashed using a plastic pestle in a 1.5-ml microcentrifuge tube containing 180 µl of buffer AL and 20 µl of proteinase K solution. Following incubation at 56 °C for 2 h, DNA was extracted following standard protocols. For the final step, 150 µl of buffer AE was used to elute the DNA from each sample.

#### Development of species-specific PCR primer pairs

*Tpi* sequences containing a highly variable intron, which were amplified by using the primers [26] in our previous studies ([21] and LC468358-LC468414), were subjected to multiple alignment by using the software MEGA 7 [27]. The aligned sequences were subjected to Primer-BLAST software [28] to design species-specific primers for *E. mandarina* and *E. hecabe*, respectively. According to in silico analyses, the primer pairs Em4-F and Em4-R amplifies ca. 120-bp products of *E mandarina*, and the primer pairs Eh6-F and Eh6-R amplifies ca. 375-bp products of *E. hecabe* (Table 2). These primer pairs were not considered to amplify any products from *E. blanda*. All of these primers were synthesized by FASMAC Co., Ltd. (Kanagawa, Japan).

**Table 2:** Sequences of Tpi primers used in this study

Primer	Sequence	Target species	Reference
Em4-F	5′–GGCTCCAACAATTGGGAGATTA–3′	*Eurema mandarina*	This study
Em4-R	5′–TACAGGCAATGACCTTGAGGC–3′		
Eh6-F	5′–TGTGGCCTTCTGCCCTATTAAA–3′	*Eurema hecabe*	This study
Eh6-R	5′–ACAGGCAATGACCTTGAGTC–3′		
Tpi-F	5′–GGTCACTCTGAAAGGAGAACCACTTT–3′	Universal (Lepidoptera)	Jiggins et al. [26]
Tpi-R	5′–CACAACATTTGCCCAGTTGTTGCAA–3′		

#### PCR methods

The PCR reaction mixtures consisted of 0.5 µl of genomic DNA solution, 1 µl of dNTP Mixture, 1 µl of 10 × PCR buffer, 0.5 μl of each primer, 0.05 µl of Takara Ex Taq (Takara Shuzo Co., Ltd., Kyoto, Japan), and X µl distilled water, where X was 5.45 for multiplex PCR and 6.45 for singleplex PCR. The PCR condition was 94.0 °C for 5 min, followed by 35 cycles of 94.0 °C for 30 s, 48 °C for 30 s, 72.0 °C for 30 s, and finally 72.0 °C for 7 min. The universal *Tpi* primers [26] were used as an internal positive control and distilled water was used as a negative control. The PCR products were separated using 2% agarose gel electrophoresis containing 0.01% GelRed (Wako Pure Chemical Industries, Ltd., Osaka, Japan).

### Results and discussion

As expected, a singleplex PCR using the primer pair Em4-F and Em4-R consistently amplified ca. 120-bp products from *E. mandarina* (n = 38) but no products from *E. hecabe* (n = 67) (Table 2). However, a singleplex PCR using the primer pair Eh6-F and Eh6-R consistently amplified ca. 375-bp products from *E. hecabe* (n = 67) but no products from *E. mandarina* (n = 38) (Table 2). These primer sets did not amplify any products from *E. blanda* (n = 6). Collectively, both primer pairs appeared to be suitable for species identification among *E. hecabe*, *E. mandarina*, and *E. blanda*.

**Fig. 1:**
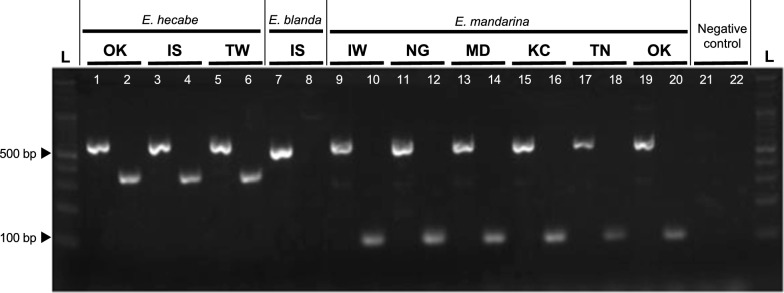
Species-specific amplification using multiplex PCR. Lanes with odd numbers are those amplified using a universal *Tpi* primer set (Tpi-F/Tpi-R) and lanes with even numbers are those amplified using multiplex *Tpi* primer sets (Em4-F/Em4-R and Eh6-F/Eh6-R). Lanes 1–6 are *E. hecabe*, lanes 7–8 are *E. blanda*, lanes 9–20 are *E. mandarina*, and lanes 21–22 are a negative control. L: 100-bp ladder. OK: Okinawa Island, IS: Ishigaki Island, TW: Taiwan, IW: Iwate, NG: Nigata, MD: Matsudo, KC: Kochi, TN: Tanegashima Island

When we performed a multiplex PCR assay including both primer pairs, Em4-F/Em4-R and Eh6-F/Eh6-R, in a PCR reaction we successfully amplified a single product of ca. 120 bp in size from *E. mandarina* (n = 38), a single product of ca. 375 bp in size from *E. hecabe* (n = 67), and no products from *E. blanda* (n = 6) (Fig. 1). Therefore, this multiplex PCR assay allows us to easily distinguish the three butterflies using a single PCR reaction.

By the advent of high-throughput sequencing, sequencing is becoming accessible to massive amounts of nucleotide sequence data, which provides reliable grounds for the taxonomic classification of different species, as well as phylogenetic inferences on different taxa [11]. However, when it comes to simple and easy methods to distinguish closely related species, multiplex PCR is still the most appropriate approach in many cases. In some cases, PCR could be substituted with loop-mediated isothermal amplification, which is easier to conduct but more difficult to design primers for [29].

In the present study, we established a multiplex PCR that can distinguish *E. mandarina* and *E. hecabe* easily, reliably, and cost-effectively. We consider that, at least in Lepidoptera, *Tpi* gene sequences are moderately variable. They are variable enough to differentiate different species but invariable enough to allow designing primers within species. Therefore, *Tpi* is a potential target for marker development of multiplex PCR to distinguish other closely related lepidopteran species when other approaches, such as mtDNA, are unavailable. Along with other nuclear genes, the *Tpi* gene is also useful for constructing a higher-level phylogeny of insects [30].

## Limitations

We mainly used *E. mandarina* and *E. hecabe* that were collected in Japan. While *E. mandarina* is distributed primarily in Japan, *E. hecabe* is widely distributed in Asia, Africa, and Australia. Therefore, the robustness of this multiplex PCR needs to be confirmed by including samples from other populations in the world, particularly for *E. hecabe*.

## Supplementary information

**Additional file 1.** Sample information used in this study.

## Data Availability

All data generated or analyzed during this study are included in this published article and its supplementary information file.

## Abbreviations

mtDNA: Mitochondrial DNA
PCR: Polymerase chain reaction
*Tpi*: Triose phosphate isomerase
